# *Fusarium oxysporum* Triggers Tissue-Specific Transcriptional Reprogramming in *Arabidopsis thaliana*

**DOI:** 10.1371/journal.pone.0121902

**Published:** 2015-04-07

**Authors:** Rebecca Lyons, Jiri Stiller, Jonathan Powell, Anca Rusu, John M. Manners, Kemal Kazan

**Affiliations:** 1 CSIRO Agriculture Flagship, Queensland Bioscience Precinct, Brisbane, QLD, Australia; 2 CSIRO Agriculture Flagship, Black Mountain Laboratories, Canberra, ACT, Australia; 3 Queensland Alliance for Agriculture & Food Innovation (QAAFI), The University of Queensland, St Lucia, Brisbane, Queensland, 4067, Australia; Instituto de Biología Molecular y Celular de Plantas, SPAIN

## Abstract

Some of the most devastating agricultural diseases are caused by root-infecting pathogens, yet the majority of studies on these interactions to date have focused on the host responses of aerial tissues rather than those belowground. *Fusarium oxysporum* is a root-infecting pathogen that causes wilt disease on several plant species including *Arabidopsis thaliana*. To investigate and compare transcriptional changes triggered by *F*. *oxysporum* in different Arabidopsis tissues, we infected soil-grown plants with *F*. *oxysporum* and subjected root and leaf tissue harvested at early and late timepoints to RNA-seq analyses. At least half of the genes induced or repressed by *F*. *oxysporum* showed tissue-specific regulation. Regulators of auxin and ABA signalling, mannose binding lectins and peroxidases showed strong differential expression in root tissue. We demonstrate that *ARF2* and *PRX33*, two genes regulated in the roots, promote susceptibility to *F*. *oxysporum*. In the leaves, defensins and genes associated with the response to auxin, cold and senescence were strongly regulated while jasmonate biosynthesis and signalling genes were induced throughout the plant.

## Introduction

Plants are constantly exposed to microbes and pathogens and must coordinate tightly regulated immune responses to ensure their survival and reproductive success. The rhizosphere is a rich source of microorganisms, and some of the most destructive plant diseases are caused by root infecting fungal or oomycete pathogens such as *Fusarium*, *Rhizoctonia*, *Pythium* and *Phytophthora* species. Although these pathogens infect root tissues, visible disease symptoms often appear in above-ground parts of the plant. Therefore, most studies to date have focused on the response of aerial tissue rather than the roots to such pathogens [1]. Since roots and shoots are normally exposed to different microorganisms, it has been suggested that the two tissues may have evolved defence strategies that differ in amplitude, timing or specificity [2]. However, there is a scarcity of studies comparing the response of roots and shoots to root-infecting fungal pathogens to directly test these hypotheses.

*Fusarium oxysporum* is a ubiquitous root-infecting fungal pathogen that causes wilt disease on several plant species including *Arabidopsis thaliana*. *F*. *oxysporum* is considered a hemibiotrophic pathogen because it begins its infection cycle as a biotroph but later changes to a necrotroph. In the biotrophic phase, *F*. *oxysporum* establishes infection via the roots and travels towards the vasculature. Upon perception of fungal elicitors, plants mount a basal defence response (PTI) characterised by an oxidative burst, cell wall callose deposition and transcriptional changes which is designed to inhibit microbial colonisation. Successful pathogens such as *F*. *oxysporum* have evolved mechanisms to overcome this relatively weak defence response and colonise the plant [3]. Once in the vasculature, *F*. *oxysporum* travels upwards in the plant and accumulation of fungal mycelia and defence related compounds in the xylem cause vascular wilting. As the infection progresses, *F*. *oxysporum* changes to a necrotrophic pathogen, causing foliar necrosis, lesion development and eventual plant death. *F*. *oxysporum* is thought to secrete phototoxic compounds which cause root cell collapse and veinal chlorosis in the leaves and is also proposed to reprogramme the host to induce senescence and facilitate disease during the necrotrophic phase of infection [4, 5]. Fungal effectors such as secreted-in-xylem 4 (SIX 4) promote infection most likely by promoting host JA responses [6]. Interestingly, the jasmonate (JA) signalling pathway both negatively and positively contributes to *F*. *oxysporum* resistance in *A*. *thaliana*. *F*. *oxysporum* produces bioactive jasmonates which presumably promote host senescence [7] while the JA receptor mutant *coi1* exhibits extreme resistance to *F*. *oxysporum*, suggesting that *F*. *oxysporum* hijacks the host JA signalling pathway to facilitate disease [8]. Adding to the complexity, JA-dependent defences may also have a relatively small but significant effect on this pathogen [9]. Other phytohormone pathways have also been shown to regulate the host response to *F*. *oxysporum*. Ethylene and auxin are both thought to negatively regulate *F*. *oxysporum* resistance since the ethylene receptor mutant *etr1-1* and auxin signalling and transport mutants show increased resistance to *F*. *oxysporum* [10, 11]. The stress hormone ABA also promotes susceptibility to this pathogen [12]. Salicylic acid promotes resistance to *F*. *oxysporum*, presumably acting in the biotrophic phase of infection, although this seems to be somewhat isolate-dependent [7, 13–15].

In this study, we comparatively analysed the below- and above-ground defence responses occurring in *A*. *thaliana* in response to infection by *F*. *oxysporum*. Transcriptional reprogramming by *F*. *oxysporum* differed considerably depending on the tissue and timepoint after infection.

## Materials and Methods

### Plant materials and growth conditions, inoculation, tissue collection and disease assessment

*A*. *thaliana* Col-0 seeds were used for RNA-seq analyses. The T-DNA insertion lines *arf1-5* (SALK_079046), *arf2-6* (CS24600), *arf2-8* (CS24602) and double mutant *arf2-6/arf1-3* (CS24631) have been previously described [16–18]. T-DNA insertion mutants *prx33-1* (SALK_056847C) and *prx33-2* (SALK_062314C) were obtained from the Arabidopsis Biological Resource Centre (ABRC).

Inoculations for tissue collection and disease assessment were carried out as follows: seeds were placed on damp soil and imbibed for two days at 4°C to synchronise germination. Plants were then grown under short day conditions (8h photoperiod) at 21°C for four weeks. The *F*. *oxysporum* isolate used in this study was strain Fo5176 obtained from Dr Roger Shivas, Queensland Plant Pathology Herbarium, Brisbane, Australia. Inoculations were performed as described previously [12]. Briefly, roots of 4-week-old plants were uprooted and then dipped either in water for the mock inoculation or in a *F*. *oxysporum* suspension containing 1 × 10^6^ spores ml^-1^, replanted and placed under long day growth conditions (16 h photoperiod) at 28°C. At 24 hours and 6 days after inoculation, root and leaf tissue (three independent biological replicates, each containing a pool of ten plants) was harvested and frozen in liquid nitrogen.

Disease was measured by visually assessing symptom development on the leaves at 14 dpi either using a scale of 0–5 with 0 being asymptomatic and 5 being dead as described previously [9] or by percentage of diseased leaves [19]. At least 16 plants were assessed per line.

### *In-vitro* plant growth and *F*. *oxysporum* inoculations

*In-vitro F*. *oxysporum* inoculation assays were performed as described previously [20]. Sterilised seeds were placed on 1x Murashige and Skoog (MS) agar supplemented with 1% sucrose and incubated at 4°C for two days, then grown under short day conditions (8h photoperiod) at 21°C. The roots of two-week-old seedlings were dipped in either a *F*. *oxysporum* suspension containing 1 × 10^6^ spores ml^-1^ or water under sterile conditions, placed onto sucrose-free 1xMS agar plates and returned to short day growth conditions. At 20 dpi, the number of lateral roots arising from the primary root was counted in a 2cm region starting 0.5cm down from the hypocotyl. Root length was manually measured at 20 dpi. At least 25 plants were assessed per line.

### RNA-seq analysis

Total RNA from mock and *F*. *oxysporum*-infected tissue was extracted and DNAse treated using the RNeasy mini kit (Qiagen) according to the manufacturer’s instructions. RNA integrity was confirmed using the Agilent 2100 bioanalyser Plant Nano system (Agilent Biotechnologies). Library preparation and sequencing were performed by the Australian Genome Research Facility (AGRF). Messenger RNA was selected using Poly-A tail selection prior to preparation of 50bp single end read libraries. Sequencing was performed on an Illumina HiSeq 2000 system generating approximately from 6.5 to 16 million raw RNA-seq reads per sample.

Differential expression analysis was performed using the Tuxedo analysis suite [21]. Briefly, Bowtie2 along with Tophat were used to align generated reads to the TAIR10 *A*. *thaliana* reference genome. After expressed transfrags were assembled, Cufflinks was used to quantify gene abundance and transcriptome assemblies were then merged using Cuffmerge. To identify genes differentially expressed by *F*. *oxysporum*, Cuffdiff was performed on the following comparisons: *F*. *oxysporum* inoculated roots 1 day post inoculation (dpi) versus mock inoculated roots 1dpi (FR1/MR1); *F*. *oxysporum* inoculated leaves 1 dpi versus mock inoculated leaves 1dpi (FL1/ML1); FR6/MR6 and FL6/ML6. Statistical analysis was performed within the Cufflinks analysis with false discovery rate and correction for multiple comparisons applied using standard run parameters. Genes considered differentially expressed showed a statistically significant difference in expression values (P<0.05). 0.6–2.2% of reads did not map to the *A*. *thaliana* genome. Reads that did not align to annotated transcripts were omitted from the analysis. For reads that mapped to two transcripts, the least significantly aligned transcript(s) were omitted.

To determine the tissue or timepoint specificity of DEGs, venn diagrams and gene lists were obtained using http://genevenn.sourceforge.net/. To determine functionality of DEGs, genone ontology (GO) enrichment analysis was performed using http://bioinfo.cau.edu.cn/agriGO/ [22]. Sequence data are available from NCBI under Sequence Read Archive (SRA) accession SRP052276.

### *F*. *oxysporum* RT-qPCR

First-strand cDNA was synthesized from 1μg RNA using SuperScript RNA H- Reverse Transcriptase (Invitrogen) and oligo (dT) primer, according to the manufacturer's instructions. RT-qPCR products were amplified in 10μl reactions containing 2μl cDNA, 2x SYBR® Green PCR Master Mix (Applied Biosystems) and 0.3 pmol primers. Plant actin (AT3G18780) was quantified using primers qACT2-F and qACT2-R [23] and *F*. *oxysporum β-tubulin* was quantified using primers Foβ-Tub-F and Foβ-Tub-R [9] using the following cycling conditions: 50°C for 2 mins, 95°C for 10 mins, then 40 cycles of: 95°C for 15 sec, 60°C for 40 sec. Quantities were determined against standard curves from cDNA derived from plant or *in-vitro* grown *F*. *oxysporum* using ViiA7 software (version 1.2; Agilent Technologies). Three biological replicates and 3 technical replicates were assessed per sample.

## Results and Discussion

**Fig 1:**
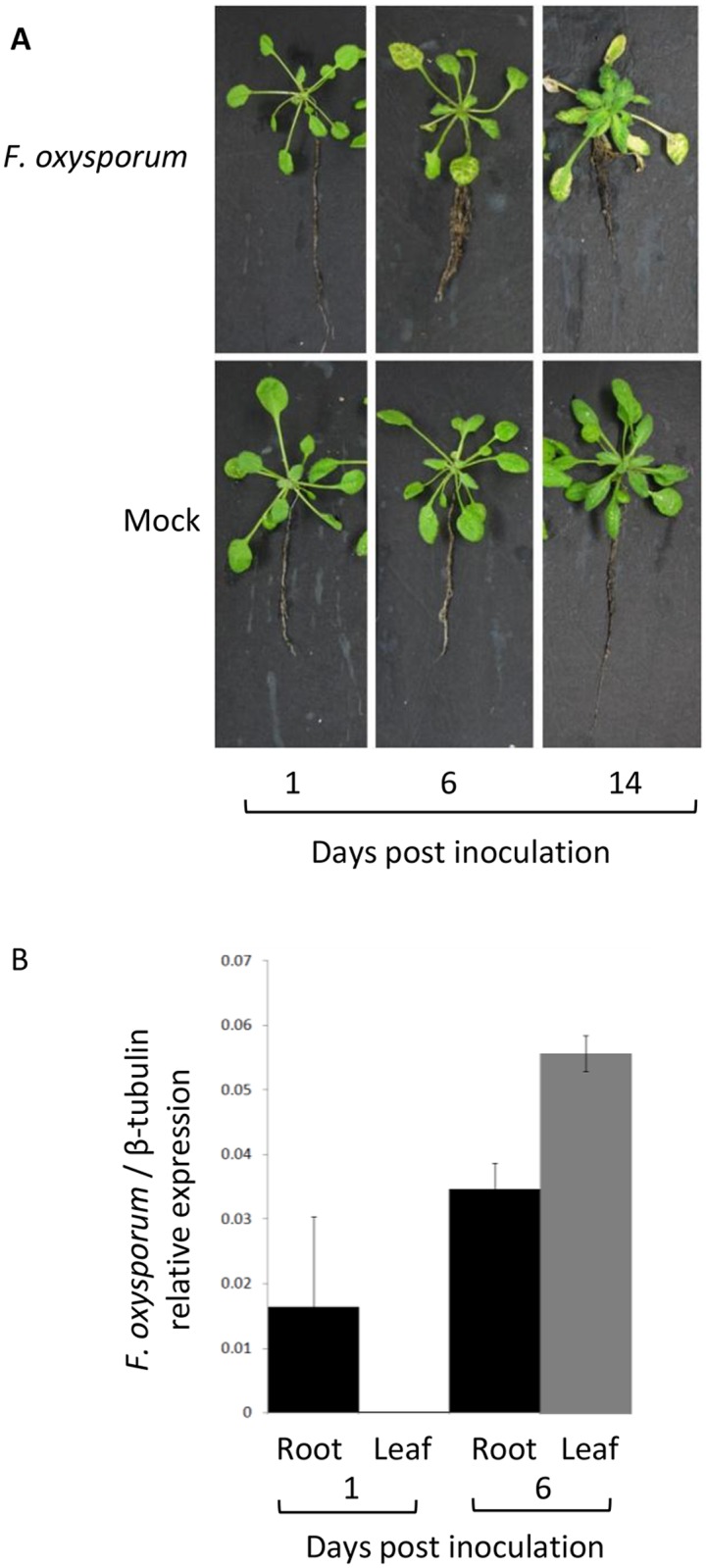
Disease symptoms and detection of *F*. *oxysporum* RNA in *A*. *thaliana* plants after *F*. *oxysporum* infection. (A) Representative plants were uprooted and photographed at 1, 6 or 14 days after *F*. *oxysporum* or mock inoculation. (B) Relative expression of *F*. *oxysporum* β-tubulin normalised to *A*. *thaliana* actin in root (black) and leaf (grey) tissue. Data show mean relative expression and standard error from 3 biological replicates containing pools of 10 plants.

To compare the response of *A*. *thaliana* roots and leaves to *F*. *oxysporum* infection, we performed RNA-seq experiments and identified genes significantly induced or repressed by *F*. *oxysporum* in either tissue at 1 or 6 days post inoculation (dpi). We used 1 dpi as an early timepoint when *F*. *oxysporum* is presumably in a biotrophic infection phase and 6 dpi when *F*. *oxysporum* is undergoing the switch from a biotrophic to necrotrophic lifestyle. At 1 dpi, *F*. *oxysporum*—inoculated plants were indistinguishable from mock—inoculated controls and *F*. *oxysporum* RNA was detectable in the roots but not in the leaves. By 6 dpi, classic disease symptoms including vein yellowing and chlorosis appeared on the leaves of Col-0 plants and fungal DNA and RNA was detectable in both root and leaf tissue (Fig 1).

### Tissue and infection stage—specific transcriptional changes are distinct

**Fig 2:**
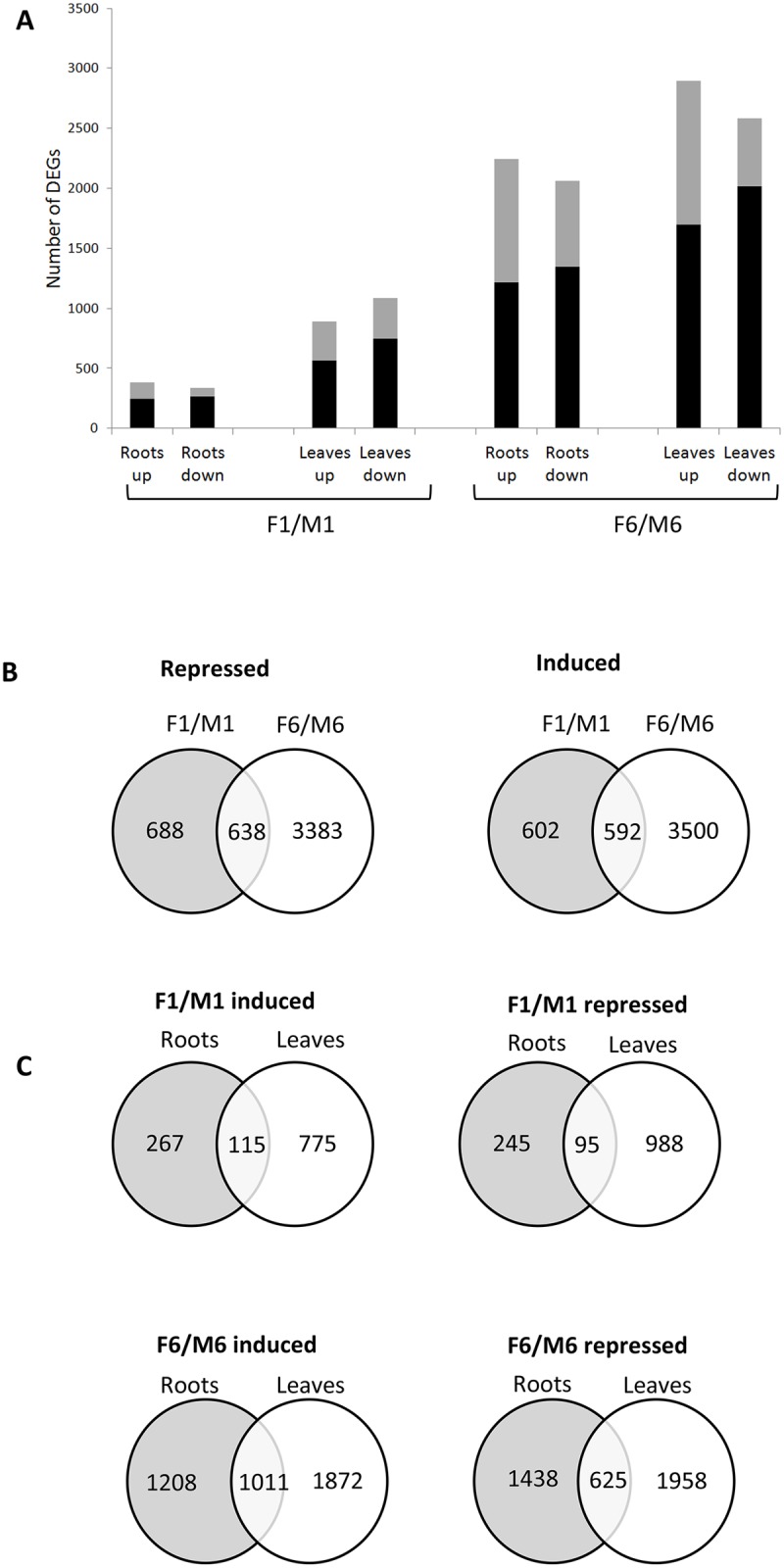
Genes induced or repressed in different tissues at early and late timepoints after *F*. *oxysporum* infection. (A) The number of genes induced (up) or repressed (down) by *F*. *oxysporum* in roots and leaves at 1 or 6 dpi. Black bars represent genes regulated <2 fold; grey bars represent genes regulated > 2 fold. (B) Overlap in *F*. *oxysporum*—responsive genes between two timepoints, 1 (F1/M1) and 6 dpi (F6/M6). (C) Overlap in *F*. *oxysporum*—responsive genes between roots and leaves.

For differential expression analysis of RNAseq data, we used the cuffdiff analysis tool and found substantial numbers of genes, ranging from 417 to 4089, were regulated by *F*. *oxysporum* depending on the timepoint, in roots and leaves. Within each timepoint, similar numbers of genes were repressed as were induced. More genes were regulated in the leaves than in the roots at both timepoints and substantially more genes were regulated at 6 dpi relative to 1 dpi (Fig 2A).

#### Comparison of gene regulation in infection timepoints

We compared the genes regulated at 1 dpi to the genes regulated at 6 dpi. Approximately half of the genes regulated by *F*. *oxysporum* at 1 dpi and 14% of the genes regulated at 6 dpi were regulated at both timepoints (Fig 2B). Secondary responses associated with hormone feedback and crosstalk may be occurring to regulate plant growth once infection has been established, accounting for the greater number of DEGs at 6 dpi. In addition, transcriptomic responses to both biotrophic and necrotrophic growth are likely to be occurring during this transitional phase of infection.

Among the genes that were regulated at both timepoints, the fold induction or repression was higher at 6 dpi than at 1 dpi (S1A Fig). The greater amplitude of regulation at the later timepoint in the leaves may be explained by the lack of direct foliar contact with the fungus at 1 dpi compared to 6 dpi. Innate immunity responses generated at 1 dpi may be increased in amplitude to more strongly defend the plant once robust infection has been established at 6 dpi.

#### Comparison of gene regulation in infected tissues

We next compared DEGs found in roots versus the leaves. More genes were regulated in the leaves than the roots at 1 dpi, even though *F*. *oxysporum* RNA was undetectable in the leaves at this early timepoint. In *Brassica oleracea*, untreated shoots respond to JA within hours of root JA treatment. This systemic response is thought to be due to the reallocation of defence compounds and primary metabolites between roots and shoots [24].

At 1 dpi, 30% of induced root genes and 12% of induced leaf genes were induced in both tissues while 45% of repressed root and 8% of repressed leaf genes were repressed in both tissues (Fig 2C). At 6 dpi, 30–45 and 25–35% of genes regulated in roots and leaves, respectively, were regulated in both tissues.

Following inoculation with the ascomycete pathogen *Colletotrichum graminicola*, the roots respond more strongly than leaves, while roots and shoots show a quantitatively different response to JA treatment [24, 25]. We asked whether roots responded more strongly to *F*. *oxysporum* than the leaves by performing pairwise comparisons on the fold induction or repression of genes that were regulated in both tissues (shared DEGs). Induction of shared DEGs was of a similar order of magnitude in both tissues, while repression of shared DEGs was slightly stronger in the leaves at 1 dpi and slightly stronger in the roots at 6 dpi (S1B Fig). Therefore, in the *A*. *thaliana—F*. *oxysporum* interaction, the amplitude of the transcriptional response is similar between roots and leaves.

To examine the pervasiveness of tissue specificity, we investigated the number of DEGs that showed tissue-specific regulation throughout the experiment. Over 50% of genes that were induced specifically in one tissue at one timepoint were exclusively regulated in that tissue throughout the timecourse (S2A Fig). We next asked whether the genes that show tissue-specific induction at 1 dpi show tissue specific expression under all conditions tested in this experiment. Of the 267 genes induced specifically in the roots at 1 dpi, only 3 were undetectable in leaves in either mock or *F*. *oxysporum* inoculated tissue in this experiment, while of the 775 genes induced specifically in the leaves at 1 dpi, 7 were undetectable in roots. These findings demonstrate that tissue-specific *F*. *oxysporum* responsive DEGs are not necessarily expressed only in that tissue under normal conditions.

### Comparison with published transcriptomic studies in the *F*. *oxysporum—A*. *thaliana* interaction

We found that *F*. *oxysporum* inoculation regulated transcription of a similar subset of novel genes in soil-grown plants as in agar-grown seedlings studied previously by [20] (Table A in S1 File). By separating root and leaf tissue prior to sequencing, we were able to show that several of the novel DEGs identified by [20] were regulated in specific tissues. In addition, less than half of the genes found to be regulated by *F*. *oxysporum* in the roots by [26] were similarly regulated in our study (Table B in S1 File). The reasons for this discrepancy are unclear, but could be due to differences in the sampling time points (2 dpi in [20] versus 1 or 6 dpi in this study) or the different technologies (microarray in [26] vs RNA-seq in this study) used in the transcriptome analyses. A recent study has demonstrated that RNA-seq has greater sensitivity and accuracy than microarray analyses [27]. Weakly expressed DEGs may have been detected using RNA-seq in our study but been below the sensitivity threshold using a microarray.

### Key functional processes showing tissue specificity

**Fig 3:**
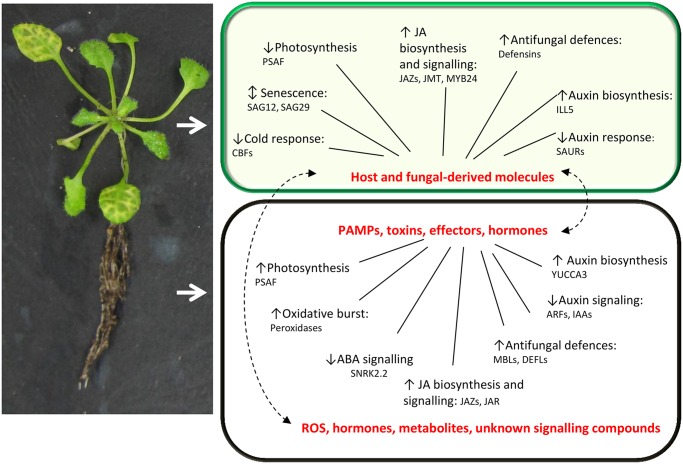
Key processes that undergo transcriptional reprogramming in response to *F*. *oxysporum* infection. Arrows indicate trend of transcriptional regulation in each of the functional categories, which were chosen based on the strength of the transcriptional response strength and the number of genes regulated. An example of a gene in each functional category is given. Upon perception of *F*. *oxysporum* in the roots, the basal defence response is elicited. *F*. *oxysporum* effectors, toxins and hormone mimics such as bioactive JAs trigger transcriptional changes and production of host-derived signalling compounds. Systemic movement of both fungal and host derived signalling molecules elicit transcriptional changes in the leaves ahead of the fungus. Transcriptional changes in both tissues are a consequence of active defence responses (i.e. production of antifungal compounds, defence signalling) *F*. *oxysporum*- driven manipulation (i.e. increased senescence in the leaves to facilitate necrotrophic infection), and the response of the plant to stress incurred by infection (i.e. changes in photosynthesis and flowering time minimise the fitness cost to the plant).

Gene ontology (GO) term singular enrichment analysis was used to identify differences in functionality between genes regulated in roots and shoots (Table C in S1 File). In general, the most significantly overrepresented functional categories were defense-related. Fig 3 summarises key processes that are transcriptionally responsive to *F*. *oxysporum* in *A*. *thaliana*. The observed induction of JA-associated genes and auxin biosynthesis genes and repression of photosynthetic genes in the leaves is consistent with trends found in previous transcriptomic studies [11, 20]. *F*. *oxysporum*-regulation of mannose binding lectins and cold-responsive genes has not been described in detail elsewhere.

#### Plant-wide processes responsive to *F*. *oxysporum* infection

Genes repressed in either tissue at 6 dpi showed an enrichment of genes associated with general biosynthesis and metabolomic processes which could be a consequence of the plant reallocating resources from development to defense. Given the established roles of JA and auxin in the *F*. *oxysporum*—*A*. *thaliana* interaction [8, 11], it could be expected that biosynthesis, signalling and transport genes in these hormone pathways may be regulated. Indeed, the majority of JA and auxin biosynthesis genes were induced in both tissues at 6 dpi. Several of the most strongly regulated auxin biosynthesis genes such as CYP83B1 and CYB71A13 catalyse the production of indole glucosinolates, which act as antimicrobial metabolites. JA signalling associated genes were also strongly induced in both tissues, with all JAZ genes induced at 6 dpi (Tables 1 and 2). The majority of JA and auxin biosynthesis genes were induced in both tissues. JA signalling associated genes were also strongly induced in both tissues, with all JAZ genes induced at 6 dpi (Tables 1 and 2).

**Table 1:** JA signalling and biosynthetic genes are induced in both tissues.

TAIR	Description	F1/M1		F6/M6	
		Roots	Shoots	Roots	Shoots
**JA biosynthesis**					
AT3G25760	ALLENE OXIDE CYCLASE 1 (AOC1)	-	-	-	8.4
AT3G25770	ALLENE OXIDE CYCLASE 2 (AOC2)	-	-	-	5.3
AT1G19640	JASMONIC ACID CARBOXYL METHYLTRANSFERASE (JMT)	-	-	-	14.3
AT1G20510	OPC-8:0 CoA ligase1 (OPCL1)	-	-	3.4	5.4
AT1G74100	SULFOTRANSFERASE 16 (SOT16)	-	-	1.5	5.1
AT2G06050	OXOPHYTODIENOATE-REDUCTASE 3 (OPR3)	-	-	3.8	9.8
AT4G16760	ACYL-COA OXIDASE 1 (ACX1)	-	-	2.3	2.0
AT3G48520	CYTOCHROME P450 (CYP94B3)	-	1.9	1.8	2.3
AT2G46370	JAR1	-	-	4.3	1.4
**JAZ**					
AT1G19180	JAZ1	1.6	-	6.2	5.6
AT1G74950	JAZ2	-	-	4.9	6.4
AT3G17860	JAZ3	-	-	3.6	1.8
AT1G48500	JAZ4	-	-	2.1	2.2
AT1G17380	JAZ5	-	1.9	9.3	8.9
AT1G72450	JAZ6	1.7	-	4.3	4.4
AT2G34600	JAZ7	-	-	3.7	7.3
AT1G30135	JAZ8	-	-	-	9.0
AT1G70700	JAZ9	-	-	5.7	5.6
AT5G13220	JAZ10	2.1	3.2	72.5	15.2
**JA-inducible defensins / antimicrobial proteins**					
AT5G24770	VEGETATIVE STORAGE PROTEIN 2 (VSP2)	2.0	-	2.0	7.8
AT5G24780	VEGETATIVE STORAGE PROTEIN 1 (VSP1)	2.1	3.1	2.0	12.2
AT1G72260	THIONIN 2.1 (THI2.1)	-	-	-	14.0
AT5G06870	POLYGALACTURONASE INHIBITING PROTEIN 2 (PGIP2)	-	-	16.0	4.0
AT1G61120	TERPENE SYNTHASE 04 (TPS04)	-	3.4	-	13.3
AT3G12500	PATHOGENESIS-RELATED 3 (PR3)	1.9	-	2.7	2.2
AT5G44420	PDF1.2	-	2.8	-	65.1
AT2G26010	PDF1.3	-	-	-	159.9
AT5G44430	PDF1.2C	-	-	-	186.0
AT2G26020	PDF1.2b	-	-	-	37.0
AT5G63660	PDF2.5	-	-	2.3	-
AT1G19610	PDF1.4	-	0.4	0.7	0.6
AT1G13609	DEFL family	-	0.5	-	-
AT2G16367	DEFL family	-	-	-	6.2
AT1G64195	DEFL family	-	0.6	-	5.2
AT2G43530	DEFL family	-	-	12.3	4.7
AT1G13607	DEFL family	-	0.5	-	3.1
AT1G76954	DEFL family	-	-	-	2.8
AT3G59930	DEFL family	-	-	2.4	2.4
AT3G05727	DEFL family	-	0.5	-	2.0
AT4G22230	DEFL family	-	-	2.5	-
AT4G22235	DEFL family	-	-	4.4	-
AT4G22212	DEFL family	-	-	6.5	-
AT4G22214	DEFL family	2.3	-	38.7	-
AT2G43510	TRYPSIN INHIBITOR PROTEIN 1 (TI1)	2.2	-	2.6	1.4

JA biosynthetic, signalling or responsive genes regulated >2 fold. Number indicates fold induction or repression by *F*. *oxysporum* relative to mock treatment.

**Table 2:** Regulation of auxin biosynthetic, signalling and transport genes.

TAIR10	Name	F1/M1		F6/M6	
		Roots	Shoots	Roots	Shoots
**Auxin biosynthesis**					
AT4G13770	CYP83A1	-	-	2.1	1.5
AT4G31500	CYP83B1 / ALTERED TRYPTOPHAN REGULATION 4 (ATR4)	-	-	-	5.1
AT2G30770	CYP71A13	2.4	-	12.3	2.0
AT1G24100	UGT74B1	-	-	1.4	3.5
AT1G04610	YUCCA 3 (YUC3)	-	-	3.0	-
AT4G28720	YUCCA 8 (YUC8)	-	-	2.4	0.3
AT2G20610	SUPERROOT 1 (SUR1)	1.4	-	-	2.4
AT5G05730	ANTHRANILATE SYNTHASE ALPHA SUBUNIT 1 (ASA1)	-	-	3.9	4.5
AT1G70560	TRYPTOPHAN AMINOTRANSFERASE OF ARABIDOPSIS 1 (TAA1)	-	-	2.3	-
AT3G44300	NITRILASE 2 (NIT2)	-	2.6	2.5	-
AT5G05590	PHOSPHORIBOSYLANTHRANILATE ISOMERASE 2 (PAI2)	-	-	-	2.2
AT1G44350	IAA-LEUCINE RESISTANT (ILR)-LIKE GENE 6 (ILL6)	-	-	9.3	5.0
AT1G51780	IAA-LEUCINE RESISTANT (ILR)-LIKE GENE 5 (ILL5)	-	-	-	30.9
AT1G51760	IAA-ALANINE RESISTANT 3 (IAR3) / JASMONIC ACID RESPONSIVE 3 (JAR3)	-	-	3.1	5.9
AT1G12200	FLAVIN MONOOXYGENASE (FMO)	1.3	0.1	-	1.4
**GRETCHEN HAGEN 3 family**					
AT1G28130	GH3.17	-	-	0.7	0.5
AT5G13370	GH3 family	2.1	1.5	2.8	
AT4G27260	WES1 /GH3.5	-	0.7	0.6	0.3
**Auxin transport**					
AT1G78100	AUXIN UP-REGULATED F-BOX PROTEIN 1 (AUF1)	-	-	0.4	0.6
AT1G22220	AUXIN UP-REGULATED F-BOX PROTEIN 2 (AUF2)	-	-	-	2.3
AT4G39403	POLARIS (PLS)	-	-	-	0.0
AT2G38120	AUXIN RESISTANT 1 (AUX1)	-	-	0.5	0.5
AT2G21050	LIKE AUXIN RESISTANT 2 (LAX2)	1.4	-	0.8	0.4
AT5G13930	TRANSPARENT TESTA 4 / CHALCONE SYNTHASE	-	2.8	-	1.5
AT3G05630	PHOSPHOLIPASE D P2	-	-	-	2.2
AT5G57090	PIN 2	-	-	2.6	-
AT1G70940	PIN 3	-	-	-	0.5
AT1G23080	PIN 7	-	-	0.6	0.5
AT1G76520	PIN-LIKES 3	-	-	2.1	1.6
AT2G17500	PIN-LIKES 5	-	-	-	4.3
AT2G14820	NAKED PINS IN YUC MUTANTS 2 (NPY2)	-	-	2.3	-
AT2G23050	NPY4	-	-	2.1	-
AT5G67440	NPY3	-	-	0.4	-
AT4G37590	NPY5	-	-	0.3	-
**Auxin/Indole-3-acetic acid (Aux/IAA) family**					
AT5G43700	IAA4	-	0.7	0.7	0.4
AT5G65670	IAA9	-	-	0.3	-
AT4G14550	IAA14	-	-	2.5	-
AT1G19850	IAA24	-	-	0.3	-
AT3G16500	IAA26	-	-	0.4	-
AT5G25890	IAA28	1.4	1.7	0.1	-
AT4G32280	IAA29	-	0.5	-	0.2
AT3G62100	IAA30	-	-	0.7	0.3
**Auxin response factors (ARFs)**					
AT5G62000	ARF2	-	-	0.4	-
AT5G37020	ARF8	-	1.5	0.4	-
AT2G28350	ARF10	-	-	0.5	-
AT3G61830	ARF18	-	-	0.5	0.8
AT1G19220	ARF19	-	-	0.4	0.8
AT4G17788	miRNA 160 targetting ARF10, ARF16, ARF17	24.2	-	0.8	55.0
AT1G31173	miRNA 167D targetting ARF6 and ARF8	-	-	-	0.6

Auxin signalling or auxin-responsive genes that are regulated >2 fold. Number indicates fold induction or repression by *F*. *oxysporum* relative to mock treatment.

#### Plant processes responsive to *F*. *oxysporum* infection in roots

The most strongly regulated genes in the roots are listed in Tables D and E in S1 File. Genes with known defensive roles were strongly regulated in the roots. For example, ER-LOCALIZED DnaJ-LIKE PROTEIN 3b (AT3G62600), a component of the ER quality control machinery involved in basal immunity [28] was strongly repressed in the roots at 1 dpi. TUBBY LIKE PROTEIN 5 (AT1G43640) promotes root colonisation by the mutualistic fungus *Piriformospora indica* [29] and was induced 73 fold in the roots at 6 dpi. Anthocyanins are suggested to play an important role in response to abiotic or biotic stress [30]. Genes involved in anthocyanin accumulation were some of the most strongly regulated genes in the roots at 1 dpi. Interestingly, of these FLS4 [31] were repressed while TRANSPARENT TESTA19 (TT19; [32]) and TT3 [33] were both induced.

#### Hormone signalling

JA and ethylene-associated genes were strongly regulated in the roots. ETO1-like, a paralog of ETO1 which represses ethylene biosynthesis was the most strongly repressed gene at 1 dpi. The second most highly-induced gene in infected roots at 1 dpi is NATA1 that encodes an ornithine N-delta-acetyltransferase involved in the production of JA-induced defensive metabolite [34] while at 6 dpi, the most responsive gene (induced 485 fold) was TAT3, a tyrosine aminotransferase responsive to JA. Several uncharacterised DEFL genes such as AT4G22214 (37 fold induction at 6 dpi) showed strong induction specifically in the roots (Table 1).

ABA is a major player in abiotic stress regulation but also contributes to defence [35]. It has been proposed that ABA may act as a root-to-shoot systemic resistance signal in some plant-microbe interactions [25]. Several ABA-associated genes were strongly regulated by *F*. *oxysporum* in the roots. At 6 dpi, the ABA receptor RCAR3 that was recently implicated in the immune response to bacteria [36] was repressed >10 fold; SNF1-related kinase2.2 (AT3G50500) which regulates the drought stress response [37] was repressed >40 fold and the ABA transporter PLEIOTROPIC DRUG RESISTANCE 12 (AT1G15520), which regulates resistance to the pathogen bacteria *Ralstonia solanacearum* [38] was induced 15 fold. At 1 dpi, AT5G35210, which encodes a PHD transcription factor required for ABI4 activation [39], was repressed >7 fold in the roots. Drought stress caused by the invasion of xylem vessels by *F*. *oxysporum* may contribute to the strong regulation of ABA-associated transcripts upon infection.

#### Mannose-binding lectins

Members of the Mannose-binding lectin (MBL) superfamily protein were among the most strongly induced genes in the roots (Table 3). AT1G52100 and AT5G35940 were induced >50 fold at 6 dpi, while AT5G28520 was induced >8 fold at 1 dpi. Although the specific function of MBLs in plant defence is currently unknown, it is thought that MBLs recognise potential invaders by binding to specific carbohydrates on foreign microorganisms [40]. Recently, a MBL from pepper was shown to mediate basal immunity and SA-mediated defences [38], while a MBL from strawberry promotes resistance to *C*. *acutatum* [41]. Ginkbilobin-2, a secreted MBL from *Ginkgo biloba*, inhibits the growth of *F*. *oxysporum* [42] and several other MBLs exhibit antifungal activity [43–46].

**Table 3:** Mannose-binding lectin superfamily (MBL) genes regulated >2 fold by *F*. *oxysporum*.

TAIR10	F1/M1		F6/M6	
	Roots	Leaves	Roots	Leaves
AT1G78820	-	-	0.4	0.6
AT3G51710	-	-	0.7	0.5
AT5G49870	-	-	2.0	-
AT5G38550	-	-	2.2	-
AT5G38540	-	-	2.5	-
AT2G25980	-	-	2.6	-
AT1G52050	0.6	-	2.7	-
AT1G52000	-	-	2.8	5.6
AT1G52060	0.6	-	3.1	-
AT1G52130	-	-	4.0	-
AT5G28520	8.8	-	4.5	-
AT1G52070	0.6	-	5.5	-
AT5G35940	-	-	56.4	-
AT1G52100	-	-	56.5	1.8
AT1G52000	-	-	2.8	5.6
AT3G16450	1.5	-	1.8	2.4

#### Oxidative burst

One of the earliest responses of roots to *F*. *oxysporum* infection is the oxidative burst [47]. 'Response to oxidative stress’ was a highly significant functional GO category of induced genes in the roots at 6 dpi (Table C in S1 File). Reactive oxygen species (ROS) act as signalling molecules to activate plant defences and are mainly generated by NADPH oxidases and apoplastic peroxidases [48]. Previous studies show that the leaf NADPHs RBOHD and RBOHF, that were induced in both tissues in this study (induced 1.7–4.4 fold at 6 dpi) have opposite effects on *F*. *oxysporum* disease development [20].

**Fig 4:**
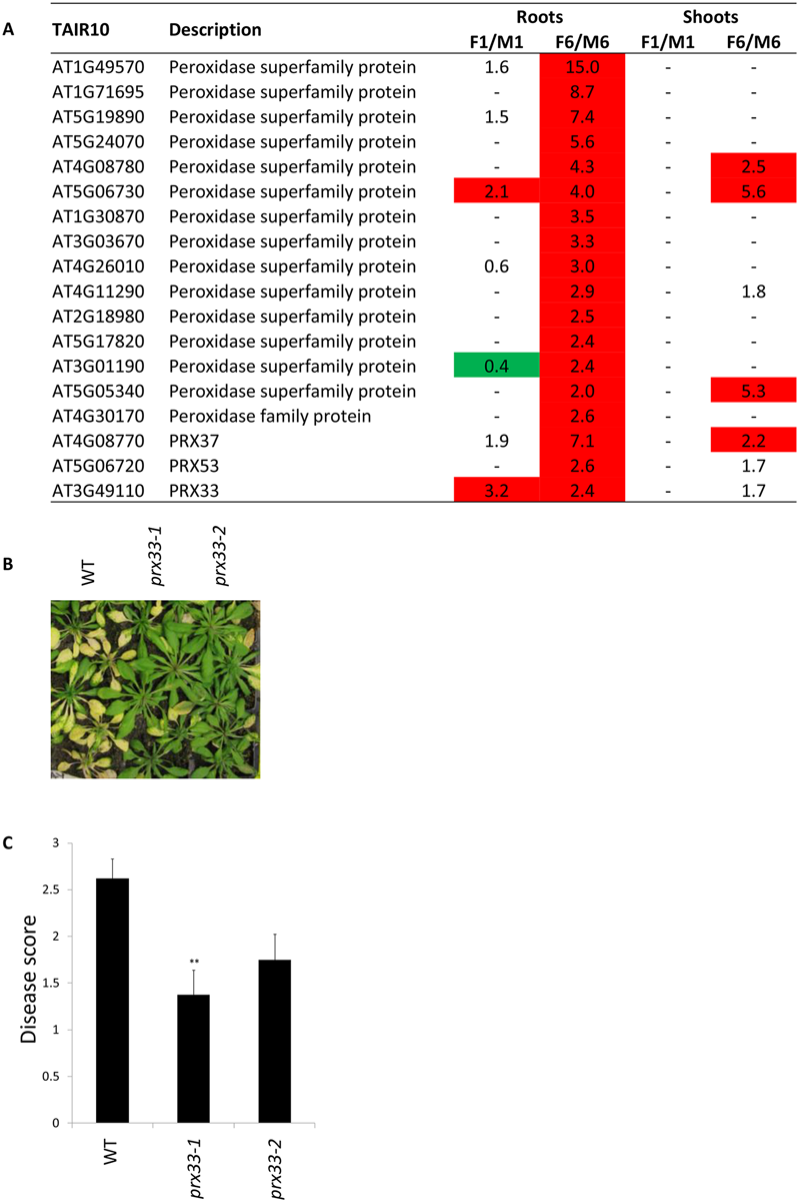
*PRX33* is induced by *F*. *oxysporum* and promotes susceptibility to *F*. *oxysporum*. (A) The fold induction or repression of peroxidases in response to *F*. *oxysporum*. (B) Representative *F*. *oxysporum-* inoculated WT and *prx33* mutant plants at 14 days post inoculation. (C) Mean disease score and standard error from 16 plants. Asterisk indicates significant difference relative to WT (P<0.01).

Numerous peroxidases were strongly induced by *F*. *oxysporum* in the roots (Fig 4A). PRX53 is JA-responsive and promotes resistance to the cyst nematode *H*. *schachtii* [49] while PRX33 is required for ROS formation in response to *F*. *oxysporum* filtrate and is a major contributor of MAMP-triggered ROS production [50, 51]. We inoculated *prx33-1* and *prx33-2*, which contain T-DNA insertions in the first intron of *PRX33* with *F*. *oxysporum* and found that they were more resistant to *F*. *oxysporum* than WT plants suggesting that PRX33 promotes susceptibility to *F*. *oxysporum* (Fig 4B and 4C). Interestingly, *A*. *thaliana* antisense or T-DNA insertion lines with reduced levels of *PRX33* and *PRX34* are more susceptible to various bacterial and fungal pathogens [50, 52] and show defective SA signaling and impaired PTI responses [53]. Numerous studies associate ROS production with defence, however recent data suggest that ROS may also promote disease development of necrotrophic pathogens. For example, *Medicago truncatula* plants with reduced ROS-producing capabilities in the roots show enhanced resistance to the root rot pathogen *Aphanomyces* *euteiches* [54]; transgenic potato plants that produce increased levels of pathogen-inducible ROS are more susceptible to *Alternaria solani* [55] while RBOHB-generated ROS promotes *Botrytis cinerea* lesion development in *Nicotiana benthamiana* [56]. In addition to basal defence signaling and modulation of defence responses such as the hypersensitive response and cell wall cross linking, ROS affect diverse physiological processes including regulation of lateral root emergence [57] and leaf senescence [58]. Indeed, *prx33/prx34* knockdown plants show delayed senescence [59]. A possible explanation for PRX33-mediated susceptibility to *F*. *oxysporum* is that that PRX33- generated ROS in the roots acts as a systemic signal to accelerate senescence in the leaves, promoting the transition from a biotrophic to necrotrophic lifestyle of *F*. *oxysporum*.

#### Photosynthesis

Interestingly, although photosynthetic genes are generally expressed at low levels in the roots of healthy plants, ‘photosynthesis’ was the most highly enriched GO category in genes induced specifically in the roots at 1 dpi (Table C in S1 File). Eighteen genes associated with photosynthesis were induced 2–3 fold in the roots at 1 dpi (Table F in S1 File). Intriguingly, 16 of these genes were repressed in the leaves at 6 dpi. Similarly to the transcriptional patterns of photosynthesis genes in our study, cotton plants exposed to drought stress show an increased transcription of photosynthesis related genes in the roots and a repression of such genes in the leaves [60] suggesting cross talk between the drought and *F*. *oxysporum* responses.

#### Host processes responsive to *F*. *oxysporum* infection in leaves

The most strongly regulated genes in the leaves are listed in Tables G and H in S1 File. The ethylene response factor RAP2.6 (AT1G43160, induced 11 fold in leaves at 1 dpi and in both tissues at 6 dpi) contributes to resistance against the beet cyst nematode *Heterodera schachtii* [61] while the glutaredoxin ROXY-2 (AT5G14070; repressed 7 fold in leaves at 6 dpi) promotes susceptibility to *Botrytis cinerea* [62].

#### Defense / antimicrobial genes

JA-associated defence genes, such as PDF1.2C and PDF1.3 (induced >150 fold 6 dpi) showed strong leaf-specific regulation at 6 dpi (Table 1). PR1, a classic marker of SA-mediated defences was induced during initial infection (4 fold up 1 dpi leaves) but was then downregulated (5 fold down 6 dpi leaves). Such a transcription pattern is typical of a hemibiotroph, whereupon SA defences elicited during the biotrophic phase are antagonised by JA signalling during the necrotrophic phase. In general, however, relatively few SA signalling genes were regulated by *F*. *oxysporum* in our experiment. One exception was SABATH methyltransferase AT3G11480 (induced 18 fold at 6 dpi in leaves), which is required for systemic acquired resistance and basal immune responses [63].

#### Photosynthesis

Consistent with published data from seedlings [20], photosynthesis was the most overrepresented functional GO category in repressed genes in leaves at 6 dpi (Table C in S1 File), which may be a consequence of pathogen-induced chlorosis and senescence. Sixty-one genes associated with photosynthesis were repressed at 6 dpi, however only 10 of these genes were repressed >2 fold (Table F in S1 File).

#### Circadian rhythm, cold response, and flowering time

Two of the most strongly regulated leaf genes modulate the circadian clock, cold response and flowering time. *FKF1*, a flavin-binding kelch repeat F-box protein and *PSEUDO-RESPONSE REGULATOR 5* were repressed >8 fold in leaves at 1 dpi [64]. Genes encoding several other cold response regulators including CBFs (CRT/DRE binding factor)/*DREB* (DRE-binding factor such as *CBF1*, *CBF2*, *COR72*, *COR413* were also strongly repressed in the leaves. Genes encoding ICE1 and ICE2 and the JAZ proteins, which mediate cross talk between cold and JA signalling [65] and SOC1 and FLC, which mediate cross talk between cold and flowering pathways [66] were all regulated by *F*. *oxysporum* in this study. Altering the transition to flowering in response to biotic stress is hypothesised to be a mechanism by the plant to maintain a balance between defence and development. Pathogen infection often results in accelerated flowering time to ensure reproductive success of the plant during stress [67].

#### Senescence

Delayed senescence has been associated with increased *F*. *oxysporum* resistance. For example, COI1, which promotes JA-triggered leaf senescence [68] is required for susceptibility to *F*. *oxysporum* [8] while *cpr5/hys1 mutants that show accelerated senescence are more susceptible to F*. *oxysporum* [5]. Whereas leaf tissue becomes necrotic and senescent in late stages of infection, roots do not show obvious signs of necrosis after infection (Fig 1A), prompting us to explore the hypothesis that senescence-associated transcriptional changes occur predominantly in the foliar tissue. At 1 dpi, plant senescence markers *SAG29* and *SAG12* were strongly induced in the leaves (>11 fold), indicating that senescence-associated responses are an important part of the infection and/or lesion development process. By 6 dpi, however, both genes were repressed >2 fold. One hypothesis to explain the marked change in SAG gene expression throughout the infection timecourse is that upon establishment of infection, *F*. *oxysporum* manipulates the host to promote leaf senescence to allow the advancing fungus to enter the necrotrophic infection phase and fully colonize the host. Once foliar infection has been established, the host might inhibit the senescence response in a counter attack to inhibit disease symptoms and fungal accumulation. *SAG29* encodes a member of the SWEET sucrose efflux transporter family proteins [69] that are associated with plant defense (reviewed by [70, 71]) while *SAG12* encodes a cysteine protease implicated in a range of senescence and cell death responses [72, 73]. Therefore the change in transcription of these genes throughout the timecourse could alternatively represent an early immune response against *F*. *oxysporum* that is inhibited by the fungus as infection progresses.

Chlorophyllase (CHL1, AT1G19670), the first enzyme involved in chlorophyll degradation, was induced 13 fold in leaves at 6 dpi. CHL1 promotes the production of ROS and resistance to the necrotrophic fungal pathogen *Alternaria brassicicola* while it negatively regulates resistance to the necrotrophic bacterial pathogen *Erwinia carotovora* [74].

### Tissue-specific regulation of auxin signalling and transport

**Fig 5:**
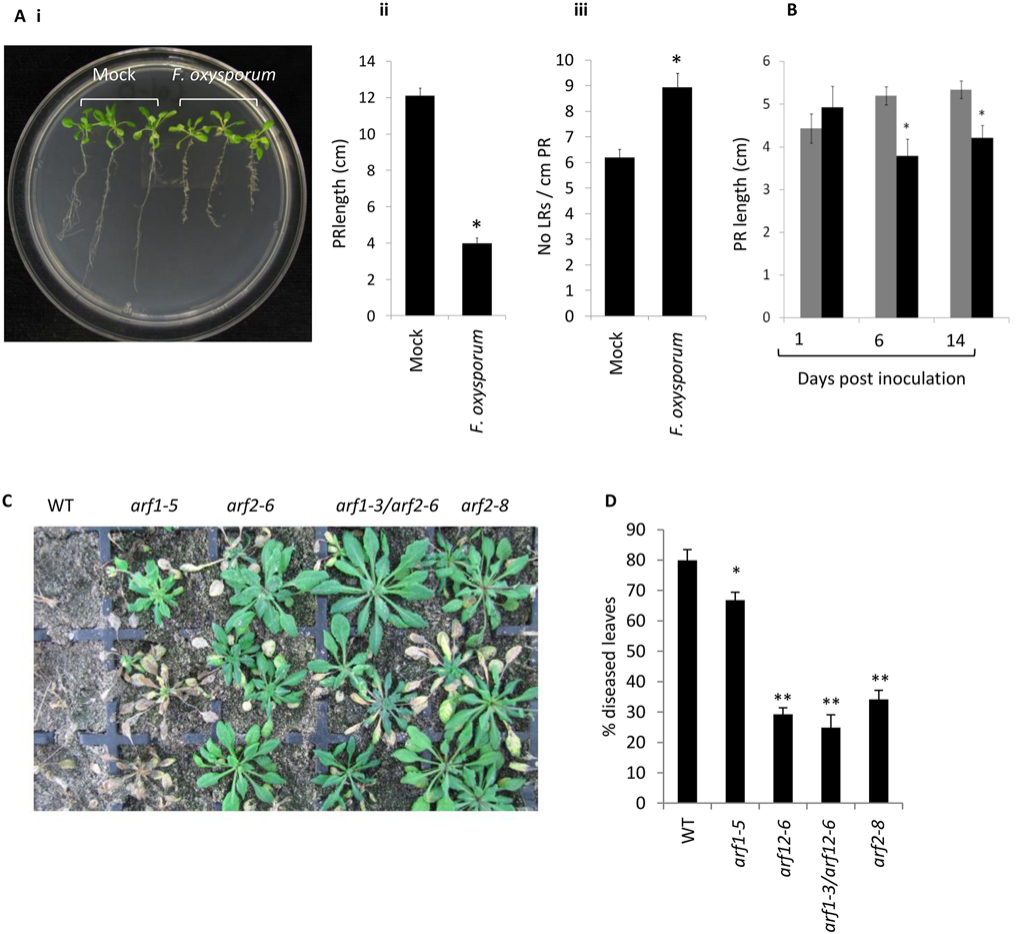
Auxin-related phenotypes and role of ARF2 in the *A*. *thaliana—F*. *oxysporum* interaction. (A) *F*. *oxysporum* inoculation triggers root growth inhibition and lateral root (LR) proliferation in agar-grown Col-0 seedlings. (i) Two week old seedlings were inoculated with water (mock) or *F*. *oxysporum* and photographed at 9 days post inoculation. (ii) Mean primary root (PR) length or (iii) mean number of LRs per cm PR in mock or *F*. *oxysporum*—inoculated agar-grown seedlings measured at 9 dpi. (B) *F*. *oxysporum* inoculation triggers root growth inhibition in soil-grown Col-0 plants. Mean PR length in mock (grey bars) or *F*. *oxysporum* (black bars)–inoculated soil-grown plants at 1, 6 and 14 dpi. Data shown are mean and standard error from >13 plants. Asterisk indicates significant difference between mock and *F*. *oxysporum* treatment (P<0.05). (C) Representative *F*. *oxysporum-* inoculated WT (Col-0) and mutant plants at 14 days post inoculation (dpi). (D) Mean percentage of diseased leaves per plant and standard error from at least 30 plants per line. Asterisks indicate significant difference relative to WT (*P<0.05; **P<0.01).

Exogenous treatment with auxin does not alter the response of *A*. *thaliana* plants to *F*. *oxysporum*, however several mutants compromised in auxin signalling and transport show increased resistance to *F*. *oxysporum*, suggesting that auxin promotes susceptibility to *F*. *oxysporum* [11, 75]. In agar-grown seedlings, a marked inhibition of root elongation and increased lateral root proliferation is evident in *F*. *oxysporum*–inoculated seedlings relative to mock inoculated seedlings (Fig 5A). Similarly, roots of soil-grown *F*. *oxysporum*-inoculated plants are shorter and appear bushier relative to mock-inoculated plants by 6 dpi (Fig 1A, Fig 5B). Such phenotypes are reminiscent of plants treated with auxin. Given that *F*. *oxysporum* enters the plant through lateral root (LR) initials [11, 76], an increased proliferation of LR might aid *F*. *oxysporum* infection. Several genes differentially expressed >2 fold in the roots, including CONSTANS-LIKE 3 (COL3), ARF19, IAA14 [77], IAA28 [78] and the chitinase-like protein CTL1 [79] regulate lateral root growth or formation. CALLOSE SYNTHASE 3 (CS3; AT5G13000) inhibits callose accumulation in emerging lateral roots [80]. Strong repression of (>8 fold) of *CS3* in the roots at 6 dpi may be a mechanism to increase physical barriers against *F*. *oxysporum* entry.

We identified genes involved in auxin signalling or transport that were induced or repressed >2 fold (Table 2). Loss of function mutants of three auxin transport regulators that are *F*. *oxysporum*-responsive: AUX1 (both tissues), PIN2 (roots) and TRANSPARENT TESTA 4 (leaves), have previously been shown to contribute to the host response to *F*. *oxysporum* [11].

#### Auxin-associated *F*. *oxysporum*-responsive genes in leaves and roots

POLARIS, which was the most strongly repressed gene in the leaves at 6 dpi (Table H in S1 File) encodes a peptide that is auxin-inducible and promotes ethylene-mediated auxin flux and biosynthesis [81]. SMALL AUXIN UPREGULATED RNAs (SAURs) are members of a large gene family containing early-responsive auxin genes of largely unknown function [82]. Thirty SAURs or SAUR-like genes were regulated >2 fold by *F*. *oxysporum* and the majority of them were repressed in the leaves at one or both timepoints (Table I in S1 File).

Four members of the five *NAKED PINS IN YUC* genes, which have been implicated in lateral organogenesis and the root gravitrophic response [83] were regulated specifically in roots at 6 dpi (Table 2).

AUXIN RESPONSE FACTORs (ARFs) are transcription factors that regulate auxin-responsive gene expression and are repressed by AUX/IAA proteins [84]. The majority of the ARFs and AUX/IAA transcripts that were differentially expressed >2 fold in this study were repressed by F. oxysporum. Moreover, several of these genes were repressed specifically in the roots (Table 2). Transcripts corresponding to ARF1 and the closely related protein ARF2 were repressed (1.8 and 2.5 fold, respectively) specifically in the roots at 6 dpi. To determine whether ARF1 or ARF2 play functional roles in F. oxysporum—A. thaliana interaction, we inoculated the previously described arf2, arf2/arf1 and arf1 mutants [16–18] with F. oxysporum. arf1 showed a slight but significant increase in resistance relative to WT plants while independent arf2 and arf1/arf2 mutants were significantly more resistant than both arf1 and WT plants (Fig 5C and 5D). These data suggest that ARF2 and ARF1 promote susceptibility to F. oxysporum.

While *F*. *oxysporum*-triggered transcriptional repression of *ARF1* and *2* occurs in the roots, it is unclear whether *arf1 and arf2*-mediated resistance occurs via the roots. ARF2 has been implicated in the control of lateral root growth [85], which might perturb *F*. *oxysporum* entry. In addition to promoting lateral organ formation and inhibiting root elongation, auxin also promotes leaf senescence [86]. *arf2* plants exhibit delayed leaf senescence and reduced transcription of senescence-inducing genes including SAG12. *arf1* loss-of-function mutants show a normal senescence phenotype, but act additively with *arf2* to delay senescence in the *arf1 arf2* double mutant [16, 87]. Delayed leaf senescence in the *arf2* mutants might delay the switch to necrotrophic growth of *F*. *oxysporum*, resulting in reduced disease symptoms.

ARF1 and ARF2 have also been shown to promote susceptibility to the biotrophic oomycete *Hyaloperonospora arabidopsidis* [88] and necrotrophic fungus *Sclerotinia sclerotiorum* [89], respectively. ARF1 negatively regulates the accumulation of glucosinolates [88] while ARF2 seems to mediate ABA signalling [89]. Altered ABA, SA or JA defence signalling due to perturbed crosstalk with auxin signalling in these mutants may also contribute to the enhanced *F*. *oxysporum* resistance phenotype.

## Conclusion

Roots and leaves play different biological roles but are dependent on each other for growth and responses to stress. In this article, we presented a comprehensive analysis of the transcriptional response of *A*. *thaliana* above-ground and below-ground tissue to *F*. *oxysporum* infection. As examples of the functional significance of our transcriptome analyses, we demonstrated that *ARF2* and *PRX33*, two genes differentially expressed in the roots, both promote susceptibility to *F*. *oxysporum*. Interestingly, both genes also promote leaf senescence, prompting us to suggest that ARF2 or PRX33 might propagate root-to-shoot signals that accelerate the transition from biotrophic to necrotrophic growth and thus symptom development of *F*. *oxysporum*. Genetic modification of PRX33 or ARF2 in crops may increase resistance to *F*. *oxysporum* in the field. However, potential benefits of such a strategy need to be considered alongside possible pleiotrophic effects such as altered flowering time, which could lead to yield penalties. Our findings demonstrate the existence of infection stage and tissue-specific transcriptional responses and enhance our understanding of the interaction between plants and hemibiotrophic root-infecting fungal pathogens. Future studies will focus on the functional dissection of genes that are differentially regulated by *F*. *oxysporum* in the roots and have not previously been shown to play a role in defence.

## Supporting Information

S1 Fig Comparison of the amplitude of response between timepoints and between tissues. The mean fold induction or repression of genes that were regulated in (A) both timepoints (middle circles, Fig 2B) or in (B) both tissues (each middle circle, Fig 2D) was compared using pairwise comparisons. Data shown are mean fold change and standard error observations. Asterisk indicates significant difference (P<0.05) in a paired T-test.
(TIF)

S2 Fig Assessing the tissue specificity of genes throughout the experiment and under basal conditions. Proportion of genes showing tissue-specific induction at one timepoint (indicated above Venn diagram) that show tissue-specific regulation throughout the time-course. The number of DEGs that are only ever regulated by *F*. *oxysporum* in the designated tissue are shown in parenthesis.
(TIF)

S1 File Supporting tables. **Table A**, Novel *F*. *oxysporum*-regulated genes identified in 2 week old seedlings [16] and their regulation in this study. **Table B**, Genes regulated by *F*. *oxysporum* in the roots of *A*. *thaliana* soil-grown plants at 2 dpi [17] and their regulation in this study. **Table C**, Results of gene ontology (GO) singular enrichment analysis (SEA comparing the top five most highly overrepresented functional gene ontology categories between tissues for each timepoint. **Table D**, The ten most strongly induced or repressed genes in the roots at 1 dpi. Highlighted values indicate a fold change >2. **Table E**, The ten most strongly induced or repressed genes in the roots at 6 dpi. Highlighted values indicate a fold change >2. **Table F**, Photosynthesis associated genes differentially regulated by *F*. *oxysporum*. Highlighted values indicate a fold change >2. **Table G**, The ten most strongly induced or repressed genes in the leaves at 1dpi. Highlighted values indicate a fold change >2. **Table H**, The ten most strongly induced or repressed genes in the leaves at 6dpi. Highlighted values indicate a fold change >2. **Table I**, *SMALL AUXIN UPREGULATED* (*SAUR*) and *SAUR-like* genes regulated > 2 fold by *F*. *oxysporum*. Highlighted values indicate a fold change >2.
(XLSX)
