# Supplementary figures and images for: Fusarium oxysporum Triggers Tissue-Specific Transcriptional Reprogramming in Arabidopsis thaliana
Source: PLoS One. 2015 Apr 7;10(4):e0121902. doi: 10.1371/journal.pone.0121902 (PMC4388846; doi:10.1371/journal.pone.0121902)

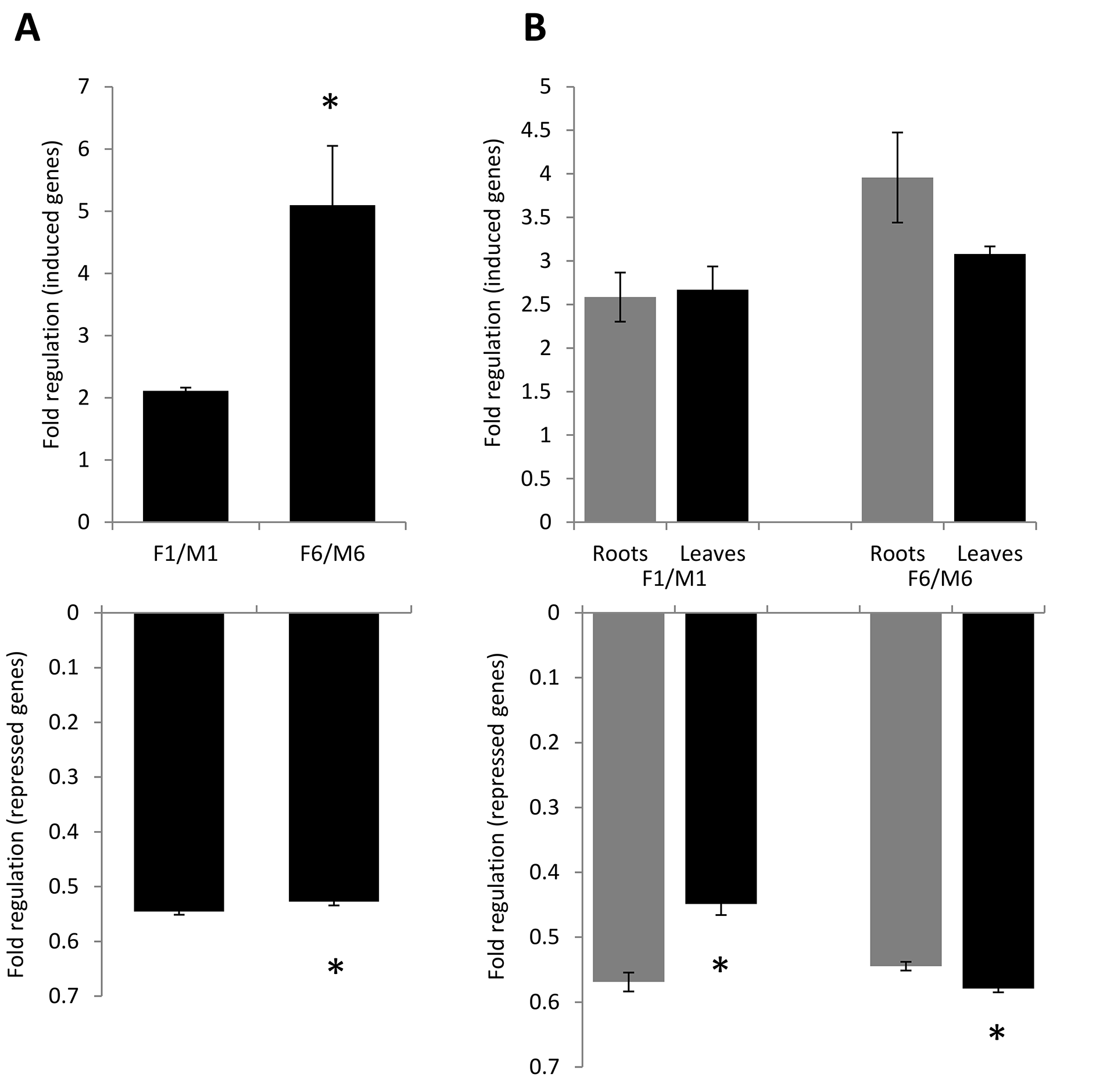

Supplement: S1 Fig — The mean fold induction or repression of genes that were regulated in (A) both timepoints (middle circles, Fig 2B) or in (B) both tissues (each middle circle, Fig 2D) was compared using pairwise comparisons. Data shown are mean fold change and standard error observations. Asterisk indicates significant difference (P<0.05) in a paired T-test. (TIF) [file pone.0121902.s001.tif]

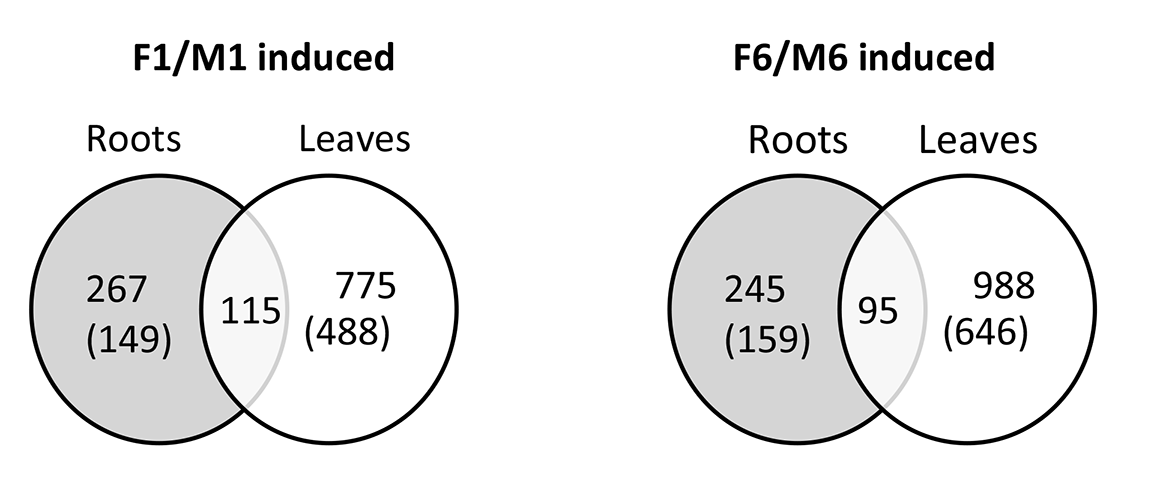

Supplement: S2 Fig — Proportion of genes showing tissue-specific induction at one timepoint (indicated above Venn diagram) that show tissue-specific regulation throughout the time-course. The number of DEGs that are only ever regulated by F. oxysporum in the designated tissue are shown in parenthesis. (TIF) [file pone.0121902.s002.tif]
